# Pulmonary Vascular Congestion: A Mechanism for Distal Lung Unit Dysfunction in Obesity

**DOI:** 10.1371/journal.pone.0152769

**Published:** 2016-04-01

**Authors:** Beno W. Oppenheimer, Kenneth I. Berger, Saleem Ali, Leopoldo N. Segal, Robert Donnino, Stuart Katz, Manish Parikh, Roberta M. Goldring

**Affiliations:** 1 André Cournand Pulmonary Physiology Laboratory, Division of Pulmonary, Critical Care and Sleep, Bellevue Hospital/New York University School of Medicine, New York, NY, United States of America; 2 Bellevue Hospital Bariatric Center, Department of Surgery, New York University School of Medicine, New York, NY, United States of America; 3 Leon H. Charney Division of Cardiology, New York University School of Medicine, New York, NY, United States of America; University of Pittsburgh, UNITED STATES

## Abstract

**Rationale:**

Obesity is characterized by increased systemic and pulmonary blood volumes (pulmonary vascular congestion). Concomitant abnormal alveolar membrane diffusion suggests subclinical interstitial edema. In this setting, functional abnormalities should encompass the entire distal lung including the airways.

**Objectives:**

We hypothesize that in obesity: 1) pulmonary vascular congestion will affect the distal lung unit with concordant alveolar membrane and distal airway abnormalities; and 2) the degree of pulmonary congestion and membrane dysfunction will relate to the cardiac response.

**Methods:**

54 non-smoking obese subjects underwent spirometry, impulse oscillometry (IOS), diffusion capacity (D_LCO_) with partition into membrane diffusion (D_M_) and capillary blood volume (V_C_), and cardiac MRI (n = 24). Alveolar-capillary membrane efficiency was assessed by calculation of D_M_/V_C_.

**Measurements and Main Results:**

Mean age was 45±12 years; mean BMI was 44.8±7 kg/m^2^. Vital capacity was 88±13% predicted with reduction in functional residual capacity (58±12% predicted). Despite normal D_LCO_ (98±18% predicted), V_C_ was elevated (135±31% predicted) while D_M_ averaged 94±22% predicted. D_M_/V_C_ varied from 0.4 to 1.4 with high values reflecting recruitment of alveolar membrane and low values indicating alveolar membrane dysfunction. The most abnormal IOS (R_5_ and X_5_) occurred in subjects with lowest D_M_/V_C_ (r^2^ = 0.31, p<0.001; r^2^ = 0.34, p<0.001). Cardiac output and index (cardiac output / body surface area) were directly related to D_M_/V_C_ (r^2^ = 0.41, p<0.001; r^2^ = 0.19, p = 0.03). Subjects with lower D_M_/V_C_ demonstrated a cardiac output that remained in the normal range despite presence of obesity.

**Conclusions:**

Global dysfunction of the distal lung (alveolar membrane and distal airway) is associated with pulmonary vascular congestion and failure to achieve the high output state of obesity. Pulmonary vascular congestion and consequent fluid transudation and/or alterations in the structure of the alveolar capillary membrane may be considered often unrecognized causes of airway dysfunction in obesity.

## Introduction

The obese state is characterized by an hyper-dynamic, high cardiac output state with an increase in blood volume in the systemic and pulmonary circulations (pulmonary vascular congestion).[1–3] Despite elevated cardiac output, evidence for subclinical cardiac dysfunction has been demonstrated by cardiac catheterization in obese subjects.[4] In addition, echocardiographic signs of ventricular dysfunction are evident in healthy obese subjects and are reversible upon weight loss.[3, 5] We have previously demonstrated that obese subjects without clinical evidence of cardiac disease demonstrated elevated pulmonary capillary blood volume which correlated with central obesity and increased total body water.[6] In a subset of these subjects, the elevated pulmonary capillary blood volume was coupled with abnormal alveolar membrane diffusion suggesting subclinical interstitial edema. In this setting of alveolar capillary leak, functional abnormalities would be expected to encompass the entire distal lung unit which includes the airways and the gas- exchange surfaces of the lung.[7]

Abnormalities in airway function have been well recognized in patients with obesity. Increased airway resistance has been attributed to reduced lung volume from mass loading and is reversible upon weight loss.[8–11] However, despite normalization of airway resistance, residual dysfunction of the distal airways may still be observed once lung volume is restored either acutely with voluntary inflation or chronically following weight loss.[12, 13] Multiple conditions may be responsible for functional abnormalities in the distal airways including airway inflammation related to metabolic syndrome and/or overlap with coexisting diseases such as asthma.[14–18]

The present study hypothesizes that in obese subjects without clinical evidence of cardiac or pulmonary disease, pulmonary vascular congestion will affect the distal lung unit as characterized by concordant abnormalities of both the alveolar capillary membrane and the distal airways. Since increased pulmonary blood volume and interstitial edema are characteristic of heart failure physiology, the present study also hypothesizes that the degree of pulmonary vascular congestion and alveolar membrane dysfunction will be related to the cardiac response to the obese state.

## Materials and Methods

The present study was funded by the Empire Clinical Research Investigator Program and was approved by our institutional IRB. The protocol included pre-operative cardiac and pulmonary evaluation obtained in 54 non-smoking obese subjects referred to the Bellevue Hospital Pulmonary Physiology Laboratory for evaluation prior to weight reduction surgery. Subjects underwent pulmonary evaluation by spirometry, plethysmography, diffusion capacity and impulse oscillometry (IOS). All subjects underwent pre-operative cardiac evaluation; in the 24 subjects where an adequate study could not be obtained, cardiac evaluation was performed by MRI. Medical Records were reviewed to determine symptoms, medical and radiographic findings. Only subjects with normal spirometry and without history of pulmonary disease, cardiac disease or obstructive sleep apnea were included in this analysis.

### Pulmonary Function Evaluation

Testing was performed in accordance with published standards (Vmax Encore, SensorMedics, Yorba Linda, CA).[19] Data included forced expiratory volume in the first second (FEV_1_), forced vital capacity (FVC), expiratory reserve volume (ERV), and inspiratory capacity (IC). Normal spirometry was defined as FEV_1_ and FVC≥80% of predicted and FEV_1_/FVC ≥70%.[20] D_LCO_ was measured by the single-breath carbon monoxide technique and related to predicted values after being adjusted for the effect of abnormal hemoglobin concentration.[21–24] D_LCO_ was partitioned into D_M_ and V_C_ as described by Roughton and Forster according to the following equation: 1/D_LCO_ = 1/D_M_ + 1/θV_C_.[6, 25–27] For this determination, 1/θ was calculated using the equation presented by Johnson et al. assuming a value for λ of 2.5.[21] This value for λ was recommended by Roughton and Forster[27] to approximate permeability of human erythrocytes. Determinations were made in triplicate at three different FiO_2_ (0.21, 0.60, and 0.80). Alveolar PO_2_ was determined at each FiO_2_ using the alveolar air equation assuming a normal arterial PCO_2_ = 40 mmHg. A correlation coefficient >0.95 between 1/D_LCO_ and 1/ θ was required to ensure accuracy of 1/D_M_.[25, 26] To evaluate abnormalities in alveolar membrane function relative to the degree of increase in pulmonary blood volume, the ratio of D_M_ to V_C_ (D_M_/V_C_) was obtained and expressed as a percent of predicted, adjusted per unit alveolar volume (VA). All values for pulmonary function and lung diffusion were referenced to standard predicted equations.[28] The percent of predicted values for diffusion were calculated for diffusion parameters expressed per unit alveolar volume.

Impulse oscillometry (IOS) was measured with the Jaeger Impulse Oscillation System (Jaeger USA; Yorba Linda, CA). To minimize the effects of reduced lung volume due to mass loading, measurements were performed under 2 conditions: tidal breathing and following a voluntary inflation to restore end expiratory lung volume to predicted FRC; this technique and the parameters analyzed have been previously described.[12] Parameters reported included resistance at oscillation frequency of 5 Hz (R_5_), frequency dependence of resistance calculated as the difference between resistance at 5 and 20 Hz (R_5−20_) and reactance at 5 Hz (X_5_). For the purpose of evaluating properties of the respiratory system, assumptions were made based on the models of DuBois et al., Otis et al. and Mead.[29, 30] R_5−20_ reflects non-uniform distribution of airflow in distal airways, and X_5_ was assumed to reflect dynamic respiratory system elastance. Data are presented as raw data and are compared to an upper limit of normal selected from prior publications to approximate 150% of published normal values.[31–37]

### Cardiac Function Evaluation

Cardiac magnetic resonance images (MRI) were obtained in 24/54 subjects using a 1.5 Tesla system with a phased array body coil (MAGNETOM Avanto, Siemens Corporation, USA). Imaging was performed in standard views from base to apex at 10 mm intervals. Electrocardiographic-gating was used to obtain ≥12 cardiac cycles per acquisition. Phase-contrast imaging was used for images of the main pulmonary artery and ascending aorta. Ventricular volumes and ejection fractions were obtained by tracing left ventricular endocardial boundaries at both end systole and end diastole. Parameters included stroke volume and heart rate. Cardiac output was derived and normalized to body surface area (cardiac index). The pulmonary artery distensibility index was computed as the difference between systolic and diastolic pulmonary artery diameter in relation to the diastolic value and expressed as a % change.

### Statistical analysis

Data were summarized as either mean and standard deviation or median and inter quartile range. The relationship between alveolar membrane function and both distal airway and cardiac function were evaluated by linear regression. Multivariate regression was performed to determine the independent predictors of D_M_/V_C_. All analyses were performed using SPSS. Statistical significance was set as a *p* value of <0.05.

The Institutional review boards of New York University School of Medicine and Bellevue Hospital approved this study, study number 11–01998. Written informed consent was obtained from the participants as approved by the Institutional Review Boards.

## Results

Demographic data of the 54 subjects are shown in Table 1. The majority of the subjects were female with a mean age was 45 ± 12 years and mean BMI was 44.8 ± 7 kg/m^2^. The distribution of obesity demonstrated predominance of central obesity (waist circumference 130 ± 17 cm, waist hip ratio 0.96 ± 0.10). Co-morbidities suggesting presence of metabolic syndrome were present in 56% of patients. Respiratory symptoms were reported by 72% of subjects; the predominant symptom was dyspnea in 56%.

**Table 1:** Subject characteristics.

*Age*	45 ± 12
*Sex*	
Male	29%
Female	71%
*Height (cm)*	
Male	174 ± 4
Female	160 ± 8
*Weight (kg)*	
Male	140 ± 22
Female	113 ± 20
*BMI (kg/m*^*2*^*)*	
Male	46 ± 8
Female	44 ± 7
*Waist circumference (cm)*	
Male	143 ± 16
Female	126 ± 15
*Metabolic Syndrome*	56%
Hyperlipidemia	43%
Hypertension	53%
Diabetes	43%
*Cell Counts*	
White Blood Cells (10^3^/μL)	8.0 ± 2.1
Eosinophils (%)	2.6 ± 2.7
*Respiratory Symptoms*	
Dyspnea on exertion	56%
Wheeze	20%
Cough	37%

Data are mean and standard deviation

Lung function data are shown in Table 2. Lung volume assessment demonstrated vital capacity (VC) in the normal range (88 ± 13% predicted) with reduction in FRC and RV (58 ± 12 and 60 ± 16% predicted respectively). By design, FEV_1_/FVC was within normal limits in all subjects. Mean values for diffusion capacity (D_LCO_) and D_LCO_ adjusted per unit VA (D_LCO_/VA) were 18.6 ± 5.7 ml/min/mmHg and 98 ± 18% predicted, respectively. Despite normal values for D_LCO_, partition into capillary blood volume (V_C_) and membrane diffusion (D_M_) demonstrated that V_C_ was normal or elevated averaging 135 ± 31% predicted while D_M_ varied from low to elevated averaging 94 ± 22% predicted. Analysis of diffusion parameters revealed no differences in subjects with and without metabolic syndrome, diabetes or hypertension (Table 3).

**Table 2:** Lung function.

*Spirometry*	
FVC (% predicted)	88 ± 13
FEV_1_ (% predicted)	85 ± 14
FEV_1_ / FVC (%)	80 ± 5
*Lung Volumes*	
TLC (% predicted)	79 ± 9
VC (% predicted)	91 ± 14
IC (% predicted)	107 ± 17
ERV (% predicted)	54 ± 27
FRC (% predicted)	58 ± 12
RV (% predicted)	60 ± 16
*Diffusion*	
D_LCO_ (ml/min/mmHg)	18.6 ± 5.7
D_LCO_ / VA (% predicted)	98 ± 18
D_M_ (ml/min/mmHg)	34.3 ± 11
D_M_ / VA (% predicted)	94 ± 22
V_C_ (ml)	65.1 ± 16.2
V_C_ / VA (% predicted)	135 ± 31
D_M_ / V_C_	0.54 ± 0.16
D_M_ / V_C_ (% predicted / % predicted)	0.73 ± 0.24

Data are mean and standard deviation

**Table 3:** Diffusion data for subjects with and without metabolic syndrome, diabetes and hypertension.

	Metabolic syndrome*		Diabetes*		Hypertension*	
	no	yes	no	yes	no	yes
	(n = 24)	(n = 30)	(n = 31)	(n = 23)	(n = 20)	(n = 34)
Diffusion						
D_LCO_ / VA	98 ± 21	99 ± 15	99 ± 20	98 ± 15	100 ± 22	97 ± 15
D_M_ / VA	97 ± 25	91 ± 20	95 ± 25	92 ± 19	99 ± 24	91 ± 21
V_C_ / VA	135 ± 34	135 ± 28	137 ± 31	132 ± 31	131 ± 35	138 ± 28
D_M_ / V_C_	0.77 ± 0.30	0.70 ± 0.19	0.73 ± 0.28	0.73 ± 0.20	0.80 ± 0.27	0.69 ± 0.22

Data are % of predicted and are presented as mean ± standard deviation.

* Differences between subjects with and without metabolic syndrome, diabetes and hypertension were not statistically different (p > 0.10).

**Fig 1:**
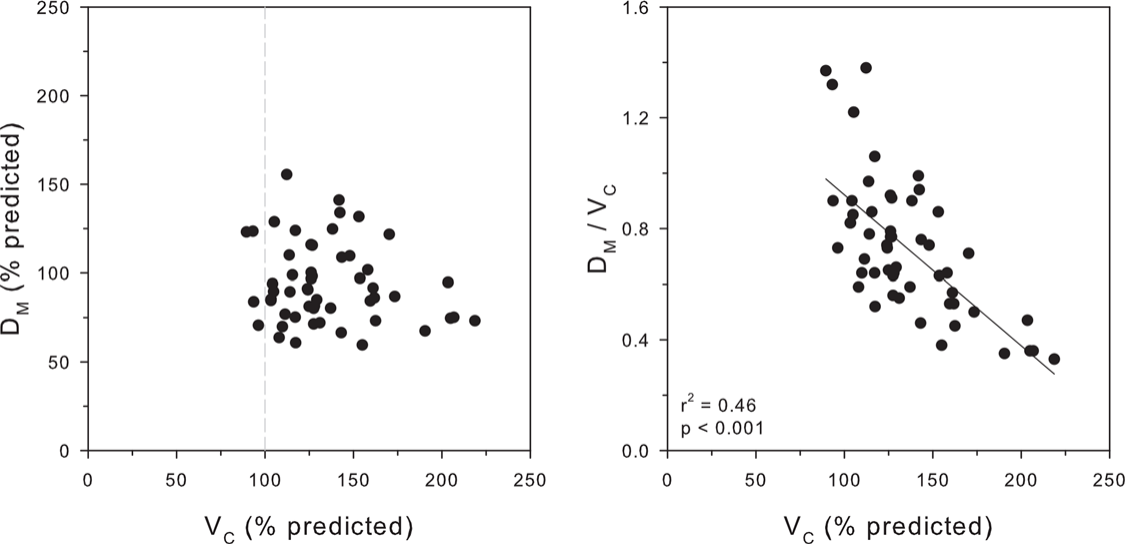
Left Panel: Relationship between V_C_ and D_M_ in individual subjects. Right Panel: Relationship between V_C_ and D_M_/V_C_ in individual subjects. Data are presented per unit alveolar volume.

The left panel of Fig 1 illustrates the relationship between D_M_ and V_C_ in individual subjects. V_C_ was above 100% predicted in all but 4 subjects with a maximal value of 219% predicted, indicating a variable degree of pulmonary vascular congestion. A wide range for D_M_ was noted from elevated values at 155% predicted, as would be expected from recruitment in the setting of elevated V_C_, to abnormally low vales at 60% predicted. There was no correlation observed between these variables (r^2^ = 0.03).

The D_M_/V_C_ was calculated in each individual as a measure of the functional efficiency of the alveolar-capillary membrane. A wide range was noted from 0.4 to 1.4 (median 0.73) with high values reflecting the expected recruitment of alveolar membrane and low values indicating that alveolar membrane function did not increase in accord with the increase in V_C_.[38–40] The right panel of Fig 1 illustrates an inverse relationship between D_M_/V_C_ and V_C_ (r^2^ = 0.46; p < 0.001). The subjects with the greatest degree of pulmonary vascular congestion demonstrated the lowest values for D_M_/V_C_.

Additional analyses demonstrated that D_M_/V_C_ was inversely correlated with age (r^2^ = 0.11, p < 0.01), but not with body size as assessed by BMI or the degree of lung compression as assessed by FRC (r^2^ = 0.004 and 0.014, respectively). Patients with respiratory symptoms and metabolic syndrome were noted across the spectrum of D_M_/V_C_. Female subjects demonstrated lower D_M_/V_C_ as compared with male subjects (0.65 vs 0.97, p < 0.01). Since all subjects had waist to hip ratio > 0.75, the influence of central obesity could not be evaluated.

**Fig 2:**
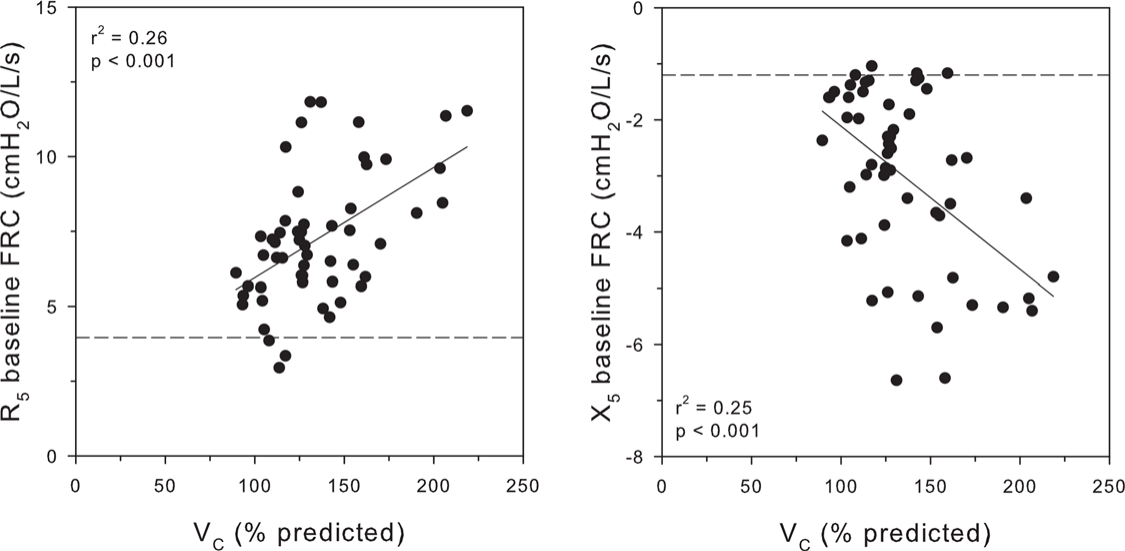
Individual values for resistance (R_5_) and respiratory system elastance (X_5_) obtained at baseline FRC are related to V_C_. The dashed lines indicate the limits of normal for each parameter.

Although all subjects demonstrated normal airway function by spirometry, abnormal respiratory function was evident by impulse oscillometry as shown in Fig 2. IOS parameters (R_5_ and X_5_) are plotted as a function of V_C_ at the baseline reduced FRC. Both R_5_ and X_5_ were linearly related to the degree of vascular congestion as expressed by V_C_ (r^2^ = 0.26, p < 0.001; r^2^ = 0.25, p < 0.001, respectively).

**Fig 3:**
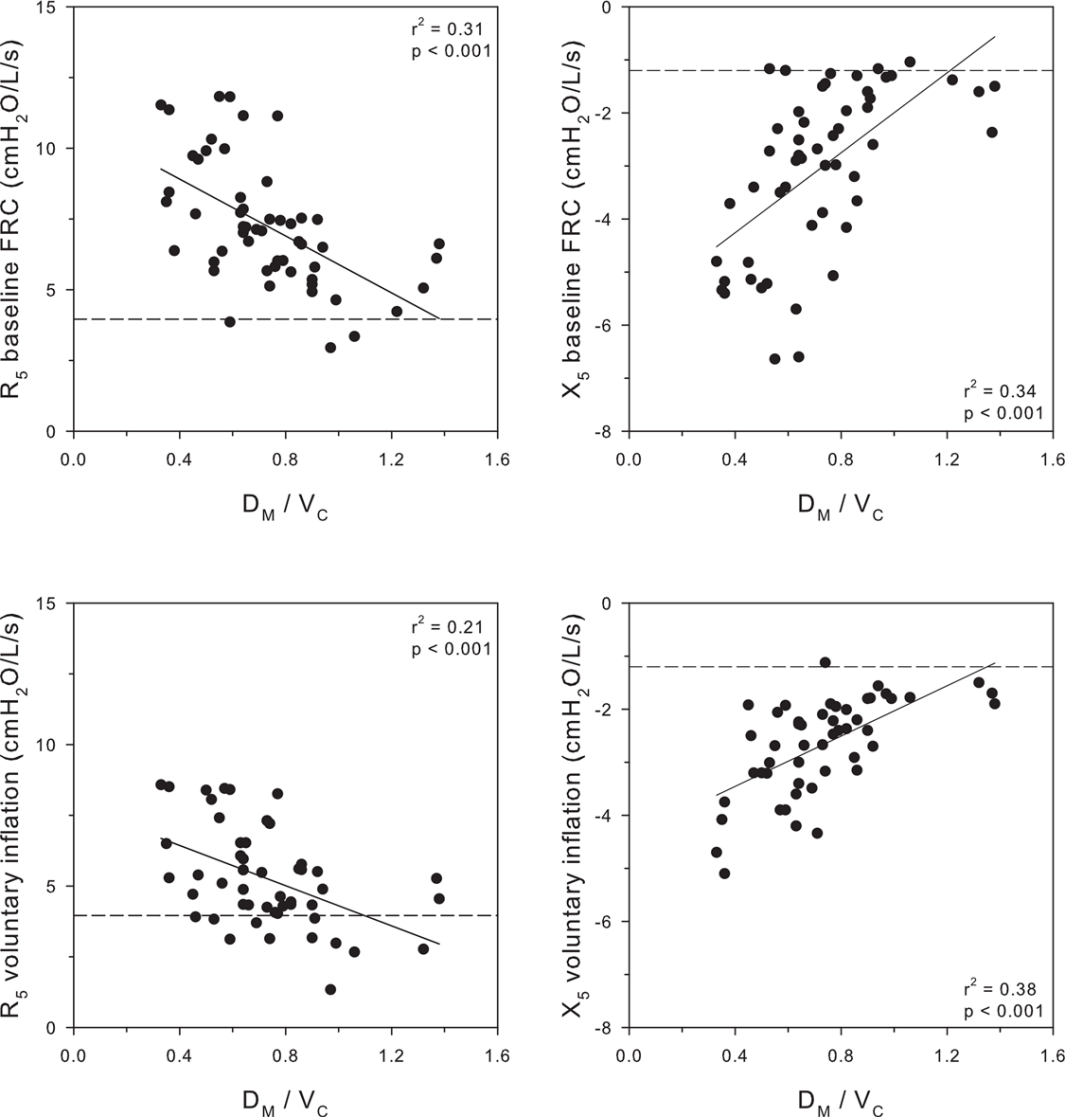
Top Panel: Individual values for resistance (R_5_) and respiratory system elastance (X_5_) obtained at baseline FRC are related to D_M_/V_C_. The dashed lines indicate the limits of normal for each parameter. Lower Panel: Individual values for resistance (R_5_) and respiratory system elastance (X_5_) obtained during voluntary inflation of end expiratory lung volume to predicted FRC are related to D_M_/V_C_. The dashed lines indicate the limits of normal for each parameter.

The concordance between abnormalities in IOS parameters and D_M_/V_C_ is shown in Fig 3. IOS parameters (R_5_ and X_5_) are plotted as a function of D_M_/V_C_ at the baseline reduced FRC and after voluntary inflation to predicted FRC. The upper figures demonstrate values obtained at baseline FRC. The most abnormal values of R_5_ and X_5_ occurred in the subjects with low D_M_/V_C_ with a significant linear correlation (r^2^ = 0.31, p < 0.001 and r^2^ = 0.34; p < 0.001, respectively). Results obtained during voluntary inflation are illustrated in the bottom figures. Voluntary inflation resulted in an increase in the end-expiratory lung volume to 94 ± 16% of the predicted FRC. While IOS values improved with inflation, the relation to D_M_/V_C_ remained unchanged (r^2^ = 0.21; p < 0.001 and r^2^ = 0.38; p < 0.001, respectively) indicating that the IOS abnormality was tightly linked to presence of alveolar membrane dysfunction and the relationship was independent of the reduction in lung volume. Moreover, both at baseline and during voluntary inflation the R_5_ was tightly linked to R_5-20_ (r^2^ = 0.69, p <0.001 at baseline; r^2^ = 0.57, p <0.001during voluntary inflation).

**Fig 4:**
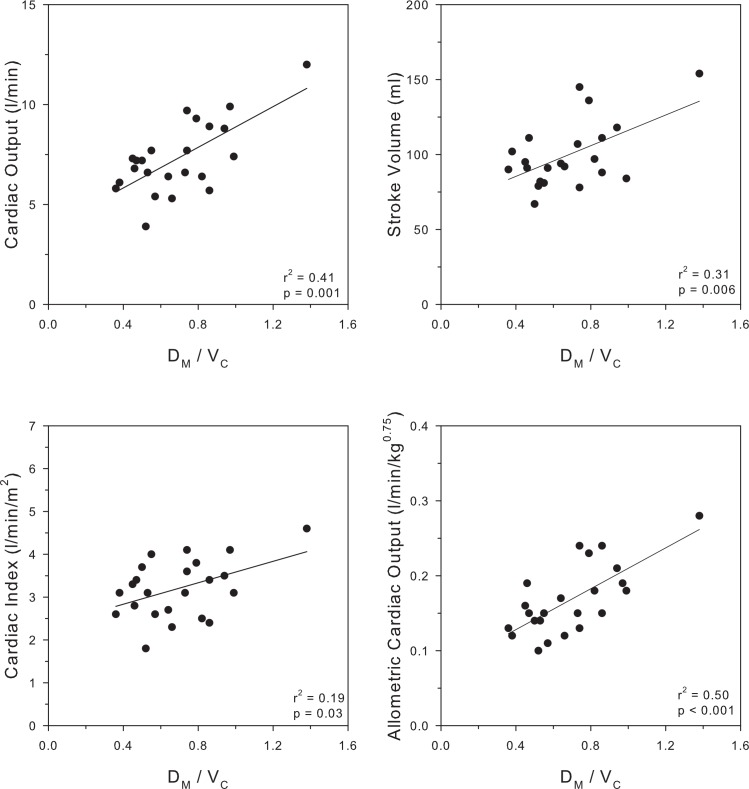
Cardiac Output, Stroke volume and Cardiac Index and Cardiac Output normalized for increasing body size based on allometric consideration (CO/kg^0.75^) for individual subjects (n = 24) are plotted against D_M_/V_C_ in 4 panels.

The relation of cardiac function obtained by MRI to the D_M_/V_C_ is explored in Fig 4. Nearly all subjects demonstrated cardiac output and stroke volume at or above the upper limit of normal for lean adults in accord with the expected hyperdynamic state of obesity. The top left panel shows a significant direct relationship between cardiac output and D_M_/V_C_ (r^2^ = 0.41, p = 0.001) and the top right panel shows a significant direct relationship between stroke volume and D_M_/V_C_ (r^2^ = 0.31, p = 0.006). The lower panels illustrates that the relationship between cardiac output and D_M_/V_C_ remained significant when cardiac output was normalized to body size either by indexing to body surface area (cardiac index, r^2^ = 0.19, p = 0.03) or indexing to published allometric considerations (cardiac output / kg^0.75^, r^2^ = 0.50, p < 0.001).[41]

**Fig 5:**
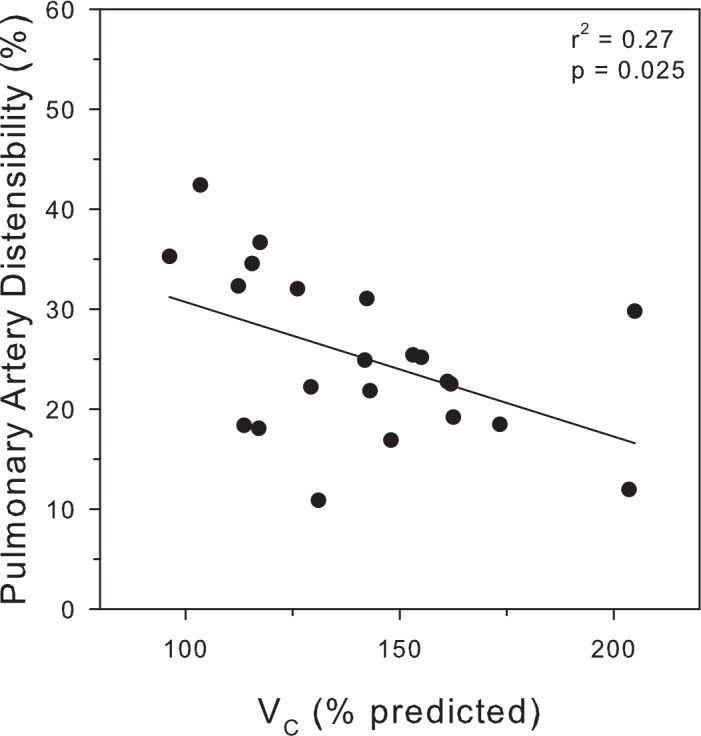
Pulmonary artery distensibility is plotted against V_C_ for individual subjects (n = 24). Data are presented per unit alveolar volume.

Cardiac function was also analyzed to evaluate left and right ventricular preload. The left ventricular end diastolic volume was essentially within normal limits at 158 ± 31 ml. In contrast, the right ventricular preload was elevated at 173 ± 41 ml (p = 0.005). Characteristics of the pulmonary vasculature are illustrated in Fig 5. Distensibility of the pulmonary artery is graphed as a function of VC. An inverse relationship was noted indicating that subjects with the greatest degree of pulmonary vascular congestion demonstrated reduced vascular distensibility (r^2^ = 0.27, p = 0.025).

## Discussion

The present study evaluated a group of obese subjects with variable degrees of pulmonary vascular congestion without clinical evidence of cardio-pulmonary disease. When pulmonary vascular congestion was associated with failure to increase alveolar membrane diffusion (low D_M_/V_C_), there was concordant distal airway dysfunction compatible with an abnormality encompassing the entire “functional unit” of the distal lung. In addition, this abnormality of the alveolar membrane was associated with failure to achieve the high cardiac output state characteristic of obesity and may therefore represent an early manifestation of cardiac dysfunction in obese subjects.

To explore the effect of an increase in pulmonary capillary volume on alveolar membrane function, the proportionality of change of D_M_ to V_C_ was evaluated by calculation of D_M_/V_C_. Geometric considerations would predict that when pulmonary vascular volume augments, the increase in V_C_ would be accompanied by capillary recruitment with consequent increase in membrane surface area (D_M_), as seen in normal individuals during exercise or in patients with ASD.[6, 39, 40, 42] However, low D_M_/V_C_ values may be seen despite elevated V_C_ when membrane function becomes compromised as in CHF.[43–45] In the present study, both patterns were observed. Subjects with high D_M_/V_C_ had appropriate membrane recruitment in accord with the increase in V_C_. Conversely, subjects with low D_M_/V_C_ had abnormal alveolar membrane function, a conclusion supported by values of D_M_ that remained either normal or were below predicted despite expansion of the pulmonary vasculature (elevated V_C_).

The presence of low D_M_/V_C_ was accompanied by greater oscillometric abnormalities. This concordance between distal airway function and D_M_/V_C_ persisted during voluntary inflation to predicted FRC indicating that it was not attributable to reduction in lung volume resulting from mass loading. The strong linear relation between the D_M_/V_C_ and the oscillometric parameters supports the notion that vascular congestion affects the alveolar membrane and the distal airways in unison thereby defining abnormality of the distal functional unit. Distal lung unit dysfunction may occur in obesity due to a number of mechanisms including: 1) compression of the airways particularly in those with central obesity; 2) alterations in membrane structure due to deposition of fat and/or collagen, increase type II cell density and increased lamellar body density; 3) changes in vascular permeability related to persistent inflammation or increased leptin; 4) airway inflammation; and 5) presence of concomitant airway disease such as asthma.[16, 18, 46–48].

The results of the present study suggest an additional mechanism for distal lung dysfunction related to pulmonary vascular congestion. We have previously demonstrated that pulmonary vascular congestion, as assessed by V_C_, correlated with increased total body water and with central obesity, a known risk factor for cardiac dysfunction.[6] This prior study also demonstrated an alteration in alveolar capillary membrane function. The present study extends these observations by demonstrating that the abnormalities in membrane diffusion are tightly linked to the magnitude of distal airway dysfunction. This linkage could be explained by vascular congestion from salt and water retention that may result in fluid transudation even in the absence of cardiac disease. However, an alternate explanation is suggested by the observed failure to demonstrate an elevated resting cardiac output as would be expected in obese subjects. Since the obesity is characterized by central vascular congestion, small changes in cardiac function may be sufficient to produce further congestion with associated fluid transudation.

A similar pattern of diffusion abnormalities has been demonstrated in patients with congestive heart failure (CHF).[43, 44, 49] In CHF, altered membrane diffusion and has been shown to be attributable to vascular remodeling as well as to fluid transudation.[43] Obese patients share features with CHF including chronically elevated pulmonary artery pressures and central congestion and, therefore, fluid transudation and/or structural changes in the pulmonary capillaries may explain abnormal membrane diffusion even in the absence of clinically evident CHF. The use of D_M_ and the D_M_/V_C_ as markers of interstitial edema has been demonstrated in athletes and confirmed by chest radiograph.[50]

Obesity has been shown to be associated with the development of a cardiomyopathy and eventually overt heart failure, conditions that become more prevalent with increasing BMI and duration of obesity.[2, 3, 51–53] An increase in circulating blood volume promotes an initial increase in stroke volume; however over time this congested state leads to increased ventricular wall stress and remodeling with subsequent ventricular dysfunction.[3] In the present study, an increase in ventricular preload was mainly evident for the right ventricle. In addition, evaluation of cardiac function by MRI demonstrated a wide range of cardiac performance. In the subjects with higher D_M_/V_C_, cardiac output and stroke volume were increased above the normal range for lean adults in accord with the hyperdynamic state of obesity. These subjects exhibited hemodynamic characteristics similar to non-obese exercising individuals who adequately increase their cardiac output in response to metabolic demands. Conversely, in the subjects with lower D_M_/V_C_, cardiac output and stroke volume remained relatively normal. This failure to increase cardiac output persisted when data were indexed to body surface area. These subjects had the highest V_C_ and, accordingly, their pulmonary arteries were found to be less distensible suggesting full recruitment and over-distension of the pulmonary capillary bed. The spectrum of cardiac performance between the groups was not dependent on body size. However, subjects with lower cardiac output and D_M_/V_C_ tended to be older, suggesting that duration of both obesity and pulmonary vascular overload may be determining factors, as has been demonstrated in prior studies.[5]

Multiple confounders need to be considered when interpreting of the results of the present study. Compression from mass loading may have influenced the results of this study. However, the effect of compression on the diffusion parameters is presumably negligible since they are measured during full lung inflation (at TLC). Similarly, the effects of airway compression on IOS parameters are unlikely to explain the observed results since abnormalities persisted when the test was performed during voluntary inflation to the predicted FRC. In addition, increasing chest wall tension during voluntary inflation would have minimal effect on the IOS data since the level of inflation was limited to the predicted FRC. This assumption is supported by the concordance between airway abnormalities and independently measured membrane diffusion even during lung inflation. An additional factor to be considered relates to the observation that there may be a relationship between gender and D_M_.[47, 54] In contrast to these previous studies, the female subjects in our study had lower D_M_ than male subjects even when adjusted for VA and compared with predicted normative data. An additional difference was that the gender difference in D_M_ was not attributable to waist to hip ratio; however, nearly all subjects had central obesity with WHR > 0.75.[54] Given the small sample size, conclusions related to gender in the present study would only be speculative.

In summary, this study demonstrates that dysfunction of the distal lung unit (alveolar membrane and distal airway) is associated with pulmonary vascular congestion and failure to achieve the hyperdynamic high output state of obesity. Pulmonary vascular congestion and consequent fluid transudation and/or alterations in the structure of the alveolar capillary membrane may be considered causes of airway dysfunction in obesity that often go unrecognized, providing further insights into the mechanisms leading to the dysfunction of the distal lung in the obese state. Further study is required to evaluate whether this physiologic phenotype may identify subjects at risk of developing a clinically overt cardiomyopathy of obesity.
